# Does spaced education improve clinical knowledge among Family Medicine residents? A cluster randomized controlled trial

**DOI:** 10.1007/s10459-020-10020-z

**Published:** 2021-01-03

**Authors:** Roland Grad, Daniel Leger, Janusz Kaczorowski, Tibor Schuster, Samara Adler, Marya Aman, Douglas Archibald, Marie-Claude Beaulieu, John Chmelicek, Evelyn Cornelissen, Bethany Delleman, Sonia Hadj-Mimoune, Samantha Horvey, Steven Macaluso, Stephen Mintsioulis, Stuart Murdoch, Brian Ng, Alain Papineau, Sohil Rangwala, Mathieu Rousseau, Teresa Rudkin, Inge Schabort, Karen Schultz, Pamela Snow, Eric Wong, Pearson Wu, Carlos Brailovsky

**Affiliations:** 1 Herzl Family Practice Centre, McGill University, 3755 Cote Ste Catherine Road, Montreal, H3T 1E2 Canada; 2 Western University, London, Canada; 3 Université de Montréal, Montreal, Canada; 4 University of Alberta, Edmonton, Canada; 5 University of Ottawa, Ottawa, Canada; 6 Université de Sherbrooke, Sherbrooke, Canada; 7 University of British Columbia, Vancouver, Canada; 8 McMaster University, Hamilton, Canada; 9 University of Calgary, Calgary, Canada; 10 University of Toronto, Toronto, Canada; 11 Queen’s University, Kingston, Canada; 12 Memorial University of Newfoundland, St. John’s, Canada; 13 Université Laval, Quebec City, Canada; 14 Family Medicine, McGill University, Montreal, Canada

**Keywords:** Education, Medical, Graduate, Family practice, Randomized controlled trial, Spaced training, Spaced education

## Abstract

Spaced education is a learning strategy to improve knowledge acquisition and retention. To date, no robust evidence exists to support the utility of spaced education in the Family Medicine residency. We aimed to test whether alerts to encourage spaced education can improve clinical knowledge as measured by scores on the Canadian Family Medicine certification examination. Method: We conducted a cluster randomized controlled trial to empirically and pragmatically test spaced education using two versions of the Family Medicine Study Guide mobile app. 12 residency training programs in Canada agreed to participate. At six intervention sites, we consented 335 of the 654 (51%) eligible residents. Residents in the intervention group were sent alerts through the app to encourage the answering of questions linked to clinical cases. At six control sites, 299 of 586 (51%) residents consented. Residents in the control group received the same app but with no alerts. Incidence rates of case completion between trial arms were compared using repeated measures analysis. We linked residents in both trial arms to their knowledge scores on the certification examination of the College of Family Physicians of Canada. Results: Over 67 weeks, there was no statistically significant difference in the completion of clinical cases by participants. The difference in mean exam scores and the associated confidence interval did not exceed the pre-defined limit of 4 percentage points. Conclusion: Further research is recommended before deploying spaced educational interventions in the Family Medicine residency to improve knowledge.

## Background and rationale

Spaced education harnesses the spacing effect and the testing effect to enhance learning in fields as diverse as Accounting and Medicine (Hussain 2019; Phillips et al. 2019). In theory, educational encounters that are spaced improve learning retention when compared with mass distribution of the same information (so-called massed learning) (Carpenter et al. 2012). This has been described as the spacing effect. An additional component of spaced education is described as the testing effect, whereby information retrieved in taking a test is better retained than information that is simply studied.

Among physicians in practice, the evidence underlying spaced education as a learning strategy was compiled in a recent systematic review (Phillips et al. 2019). This review included four studies of physicians working in the context of primary care (Gooding et al. 2017; Jiwa et al. 2014; Kerfoot et al. 2010, 2014), two of which were randomized controlled trials. Using clinical case scenarios, both trials reported improvements in knowledge retention and clinical behavior. For example, one of these trials involving physicians in primary care reported a reduction of inappropriate prostate specific antigen screening tests, as a result of online spaced education (Kerfoot et al. 2010).

In the training context, spaced education can boost knowledge retention among medical students and surgical residents (Kerfoot et al. 2007a, b). Outside of residency training in Urology, we know of four small single-site trials of spaced education in Oncology, Internal Medicine, Obstetrics/Gynecology and Pediatrics (Dolan et al. 2015; Gandhi et al. 2016; Gyorki et al. 2013; House et al. 2017). However, in searches conducted by a medical librarian in PubMed, EMBASE and ERIC, and updated to December 2019, we could find no randomized trials of spaced education in the Family Medicine residency.

We previously evaluated the resident perspective for this type of intervention in Family Medicine, as well as the feasibility of an educational trial (Grad et al. 2017; Kluchnyk et al. 2020). In 2017, we interviewed second-year residents in Family Medicine at McMaster University. In this pilot study, we sought to identify factors that could influence resident participation in an educational trial of spaced education. Alert fatigue was raised by those who reported being less enthusiastic about this type of intervention. When residents received an alert to content that was not specific to their current clinical rotation, the lower relevance of that alert was also perceived as being associated with alert fatigue.

Given the absence of trial data, whether spaced education could improve knowledge in the Family Medicine residency was an unanswered question. This was an important question for the following reasons. Foundational knowledge guides decision-making in clinical practice; yet the Canadian Family Medicine residency is the shortest among developed nations, at just two years. Furthermore, the implementation of work hour restrictions in residency reduced the time for learning in group settings. While the time to teach in residency may never be enough, the importance of foundational knowledge is supported by observational studies showing positive correlations between certification exam scores (competence) and performance in actual practice. For example, in the 1990s, family medicine residents achieving higher scores on their certification exam were more likely as family doctors in their early years of practice to prescribe fewer contraindicated drugs. While initial assessment of outcomes was limited to the first 18 months of practice, a sustained positive relationship between certification exam scores and performance over 4 to 7 years in practice was subsequently demonstrated (Tamblyn et al. 1998, 2002). However, observational studies like these, while promising, do not allow for causal inference. Given the role of primary care in health systems, the importance of rigorous trials of promising educational interventions to improve the knowledge of future family physicians is clear (Starfield et al. 2005).

We sought to provide generalizable insight into the effect of spaced education on the clinical knowledge of residents in Family Medicine. Consequently, we conducted a pragmatic trial of an intervention designed to promote spaced education via mobile app on a smartphone.

## Methods

We followed the Consolidated Standards of Reporting Trials (CONSORT) statement in reporting this cluster randomized controlled trial (Campbell et al. 2012).

### Participant flow

**Fig. 1:**
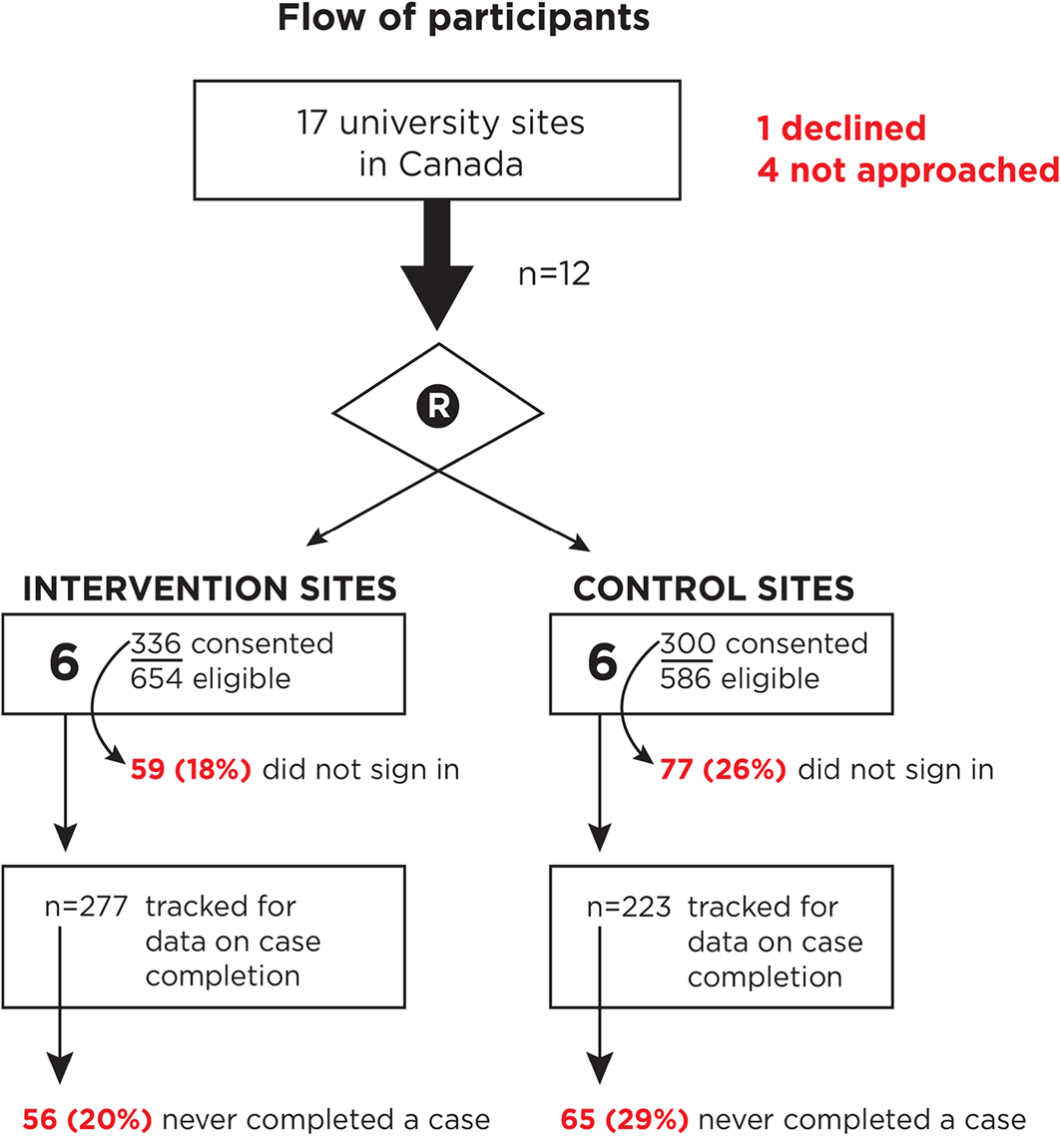
Flow diagram

The flow of participants through the trial is displayed in Fig. 1. In 2017, the principal author (RG) contacted 13 directors of university training programs in Family Medicine in Canada; 12 agreed to participate. At these sites, all residents in Family Medicine were eligible if they had commenced residency training in July 2017 and had a smartphone running Android or iOS. Residents provided informed consent to participate (online or on paper) in December 2017. The consent form explained that app usage would be tracked and their certification exam scores would be linked and then aggregated by site, after they had completed residency. Once we obtained consent, participating residents (henceforth ‘participants’) were emailed instructions on (1) How to download a free version of the app; (2) How to allow app notifications, and (3) How to enable tracking of the clinical cases they completed. Step 3 required a one-time sign-in procedure. Each participant was asked to do this, by entering their unique study ID number into their app.

### Trial design

A cluster randomized parallel group design was chosen to minimize contamination of participants. Clusters were the 12 consenting university training programs (sites) for Family Medicine residents in Canada. In December 2017, we randomly assigned these 12 sites to one of two versions of a mobile app. Through the intervention version of the app, we delivered two types of alerts to participants. The intent of these alerts was to encourage and remind participants to work their way through test questions linked to clinical cases in the app.

### Randomization

We stratified the 12 participating sites into 6 strata based on their total number of first-year residents. An expert in cluster randomized trials (JK) then used a random number generator to randomly allocate teaching sites in each pair to receive either the intervention version (n = 6) or the standard version (n = 6) of the app.

### Intervention at the cluster level

To enable this trial, we used the Family Medicine Study Guide. Launched in 2016, this app was designed to enhance and compliment the residency curriculum. At the time of the study, the app contained 75 clinical cases with up to six test questions per case. Question responses were in free-text format. On submitting a response to any question, the app then presented a textbook type answer providing immediate feedback.

**Fig. 2:**
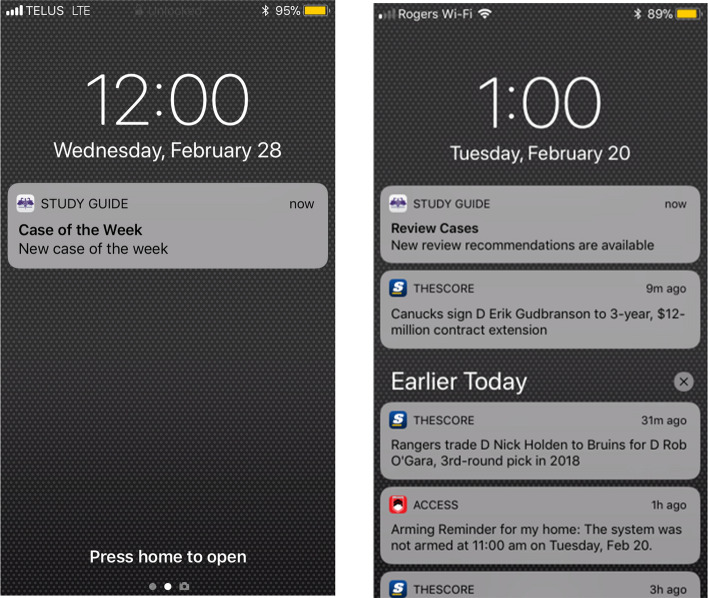
Alerts to enable spaced education as a learning strategy

We worked with the founder of the Family Medicine Study Guide to develop an intervention version of the app to enable spaced education. This version delivered two types of alerts to encourage participants to engage with the ‘Case of the Week’ (Fig. 2).

We delivered the first type of alert to the ‘Case of the Week’ on Wednesday afternoon. This alert began January 2018 and continued weekly until the certification examination in April 2019. A participant following this alert to the ‘Case of the Week’ would have completed 67 of 75 cases in the app by that date. A case was defined as ‘completed’ when a participant answered all test questions for that case, and was then listed on a page in the app titled ‘Cases to be Reviewed’. In line with previous trials of spaced education (Kerfoot et al. 2014), for each completed case we sent a second type of alert (called ‘Review Cases’) at 1- or 2-week intervals, as determined by self-rated satisfaction with case-specific answers. With this alert, we sought to remind participants to repeat cases they had already completed, to further enhance their learning. We stopped sending alerts for a clinical case when a participant was satisfied with their answers, on two separate occasions.

The control group received the standard version of the app providing the same 75 cases with test questions, on-demand. We provided no face-to-face training to use either version of the app. We sent no written material to clinician educators who supervised participating residents at any of the 12 sites.

### Outcomes

The purpose of this trial was to test whether spaced education can improve clinical knowledge as measured by scores on the Canadian Family Medicine certification examination. Given this purpose, the primary outcome was the participants’ score on the Short-answer Management Problem (SAMP) component of the 2019 certification examination of the College of Family Physicians of Canada. The College experience with SAMPs has shown them to be valid, reliable and sufficiently flexible to allow evaluation of knowledge across patient age groups and with the many different problems seen in Family Medicine (Handfield-Jones et al. 1996). Through a priori consultations with stakeholders (who were clinician educators in Family Medicine), we deemed the minimum important increase in score on the certification exam to be four percentage points. In other work, our primary outcome, certification examination score, was shown to be positively associated with performance in clinical practice (Tamblyn et al. 1998).

Our secondary outcome was the number of clinical cases completed by participants over 16-months of follow up. This outcome evaluated the extent to which our intervention encouraged engagement with clinical cases and represented a mechanism to explain any effect of spaced education on clinical knowledge.

### Sample size

We estimated sample size using scores on the certification exams from 2015 and 2016. The intra cluster correlation coefficient (ICC) was 0.052 in the year 2015 (average cluster size of university sites = 76) and 0.034 in the year 2016 (average cluster size 81). The corresponding design effects (sample size inflation factors) were calculated to be 4.9 (2015) and 3.7 (2016), respectively. The site-specific standard deviation of exam scores was 10 points for both years. From this information, an improvement of at least four percentage points corresponded to a relative effect size Cohen's d: 4 ÷ 10 = 0.4. Applying a two-sided 95% confidence interval for the difference in mean exam scores required a group sample size of 100 individuals to achieve precision of four percentage points and 80% power to reject the null hypothesis. After inflating the sample size with an expected design effect of (4.9 + 3.7) ÷ 2 = 4.4, a total sample size of 4.4 × 100 × 2 = 880 residents would be required. With an expected average cluster size of approximately 80, a total of 880 ÷ 80 = 11 clusters was needed. We sought to randomize 12 clusters to achieve a group allocation ratio of 1:1, and to account for attrition.

### Statistical methods/analyses

At the individual level, data collection on case completion began as soon as residents installed the app and signed in. We conducted intention to treat analyses using data from all 12 clusters.

For the primary outcome of clinical knowledge, the exam score was expressed as a percentage based on the absolute number of correct answers to SAMP questions. For our secondary outcome of case completion, we used Bootstrap Repeated Measurement Models to compare incidence rates of case completion between groups. Bootstrap sampling was based on samples with replacement from subjects rather than from individual data points. These analyses were performed using the statistical software R, and the R package *Hmisc* (Harrell Jr 2017). To look for clustering of case completions around the time of delivery of the weekly alert, we examined the distribution of case completion across the groups, by day of the week.

We received ethics approval first from the McGill University Institutional Review Board. Subsequently, ethical reviews were obtained from the 11 additional participating sites. The trial was registered with ClinicalTrials.gov (Identifier: NCT03312218].

## Results

At baseline, groups were well balanced at the individual level with no important differences in age, gender and iPhone ownership (Table 1). At the cluster level; the average size of the six intervention group clusters was 109, and we consented an average of 56 (range 44–95) residents per cluster. In the control group the average cluster size was 98; we consented an average of 50 (range 15–108) residents per cluster.

**Table 1:** Baseline information for each group at individual and cluster levels

	Intervention N = 335	Control N = 299
Individual level		
Age (median, 25–75%)	27 (26–30)	27 (26–30)
Women (n, %)	229, 68%	210, 70%
Men (n, %)	100, 30%	89, 30%
I prefer not to disclose (n, %)	2, 0.6%	0
I do not identify with gender binary (n, %)	4, 1.2%	0
Smartphone (% iOS)	234, 70%	213, 71%
International Medical Graduate (n, %)	40, 12%	57, 19%
Graduate degree (n, %)	80, 24%	66, 22%
Cluster level (Number/cluster)	Consented	
Site 1 (n = 167)	95, 57%	
Site 2 (n = 160)	44, 28%	
Site 3 (n = 97)	51, 53%	
Site 4 (n = 84)	56, 67%	
Site 5 (n = 76)	45, 59%	
Site 6 (n = 70)	44, 63%	
Site 1 (n = 172)		108, 63%
Site 2 (n = 106)		47, 44%
Site 3 (n = 100)		63, 63%
Site 4 (n = 90)		39, 43%
Site 5 (n = 78)		27, 35%
Site 6 (n = 40)		15, 38%

After exclusion of two ineligible persons, 500 of 634 participants completed the one-time sign-in using their unique ID number. Of the 134 (21%) who we lost to follow up (as they never signed-in), 59 (18%) were from the intervention and 75 (25%) were from the control group. 381 of the 500 (76%) participants who signed in went on to complete at least one case.

On an intention to treat basis, we linked 522 participants to their scores on the SAMP component of the 2019 certification examination of the College of Family Physicians of Canada. Consistent with no difference in the completion of clinical cases, we observed no significant effect of the intervention on clinical knowledge (Table 2).

**Table 2:** Scores of 522 participants on the spring 2019 exam

	Intervention	Control	Difference (95% CI*)
Participants	281	241	
Pass, fail	272, 9	228, 13	
Mean, SD	73.9, 4.4	73.1, 5.1	+ 0.8 (− 1.0 to + 2.6)
Min, Max	59.5, 84.0	53.9, 84.4	

*Taking an intra cluster correlation (ICC) of 0.052 into account

**Fig. 3:**
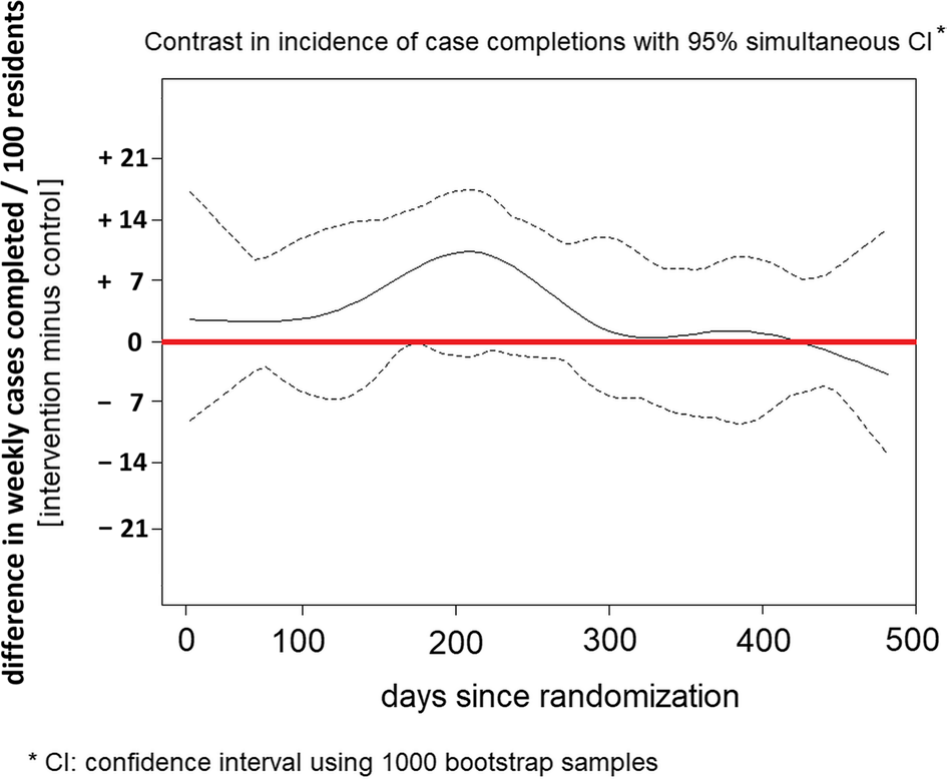
Case completion: difference between groups

With respect to our secondary outcome, a distribution of completed cases by group assignment is shown in Table 3, while the difference in case completion between groups over 16 months of follow-up is displayed in Fig. 3. In the first one-half of the follow-up period, per 100 residents in the intervention group we see a small increase of up to 7–10 cases per week in the incidence of case completion. However, at no time did the 95% confidence bands exclude a null difference. For participants in the intervention group, the occurrence of completed cases increased by an absolute value of 4.5% on Wednesday and 1.4% on Thursday, coincident with the weekly alert to a new case. Conversely, participants in the control group were 4.3% more likely to complete cases in the app on Sunday.

**Table 3:** Extent of completion of clinical cases during the trial

Average of case completions per month (cumulative total)	Intervention N = 277	Control N = 223
Zero	55 (20%)	64 (29%)
Less than 1 (1–15)	75 (27%)	50 (22%)
1 to 2 (16–32)	24 (9%)	18 (8%)
> 2 to 5 (33–80)	48 (17%)	43 (19%)
More than 5 (more than 80)	75 (27%)	48 (22%)

**Fig. 4:**
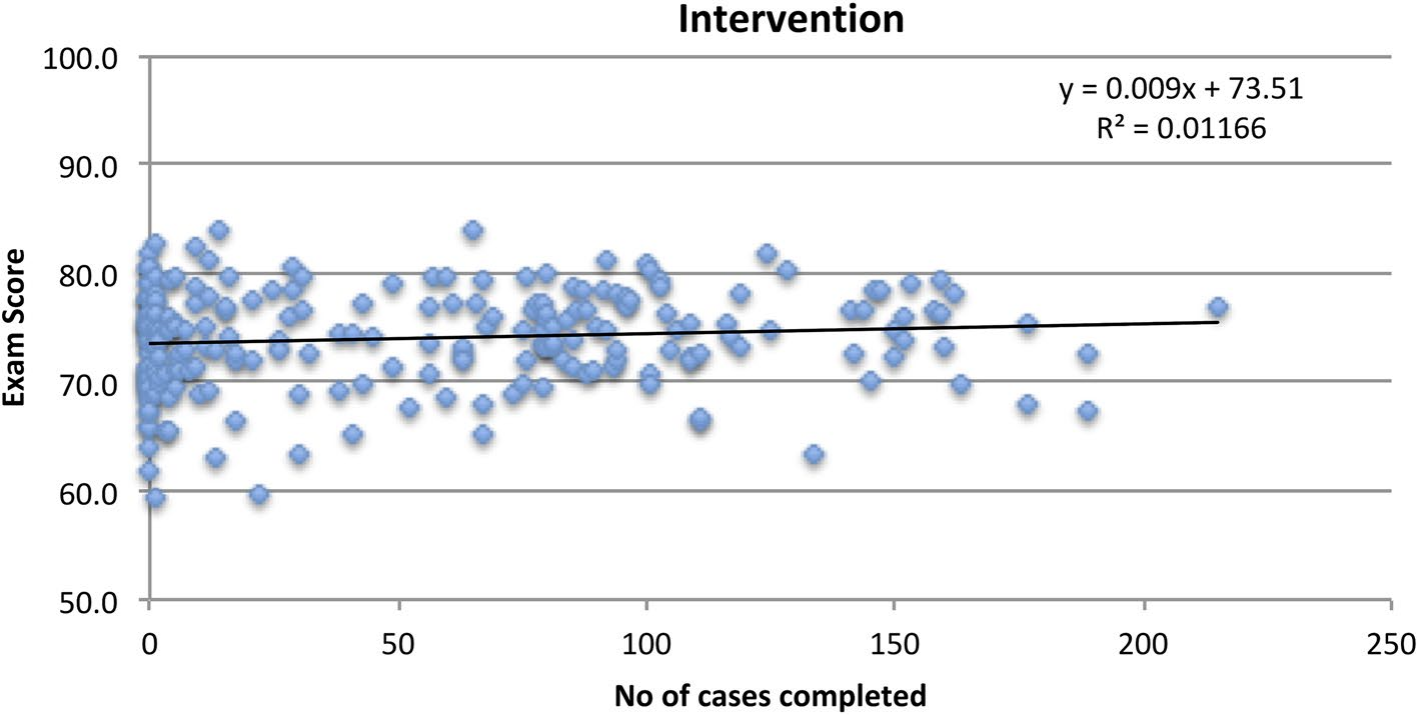
Case completion versus examination score (intervention group)

**Fig. 5:**
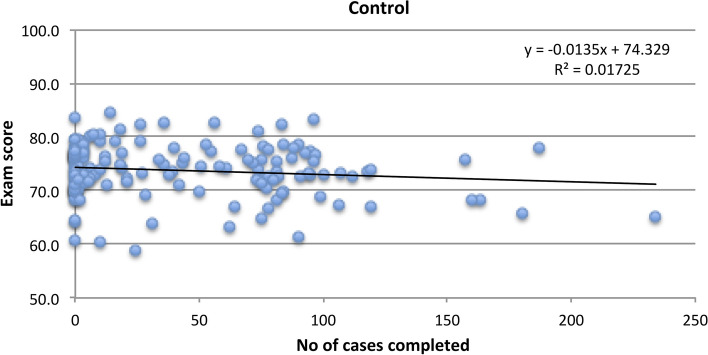
Case completion versus examination score (control group)

On a per protocol basis, we see a weak positive correlation (r = 0.11) between the number of cases completed by participants in the intervention group and their examination scores. For this same analysis in the control group, we see a weak negative correlation (r = − 0.13). There is a significant difference in the slope of these two regression lines, comparing intervention and control groups (p = 0.03) (Figs. 4 and 5).

In order to account for residual imbalances in measured covariates, we also conducted an unplanned multivariable analysis adjusting for the baseline variables of gender, exam language (English vs French) and medical school of graduation (International vs Canadian). This analysis revealed an estimated mean difference in exam scores (intervention minus control) of + 0.6, (95%CI: − 0.2 to + 2.4). Hence all analyses yielded group differences in mean exam scores and associated confidence intervals that did not exceed the pre-defined limit of practical interest of four percentage points.

## Discussion

This trial questioned whether alerts to encourage spaced education through an app improved the knowledge of residents in Family Medicine. In addition to apps, online portals and social media platforms have transformed medical education and the way that content providers deliver information. Our use of a mobile app to deliver alerts and encourage spaced education reflects current demand for educational resources that are portable, easy to use and concise.

Delivered through an app, our mobile-enabled intervention comprised both a technology dimension and a reminder dimension. The technology dimension refers to the concept of a mobile device as “the hardware and sometimes, by inference its functionality, other than SMS text-messaging” (Masters et al. 2016). A broader concept, one which includes software (apps), operating systems and the related infrastructure to support mobile device usage is referred to as mobile technologies. In clinical settings, sociotechnical barriers such as colleagues’ views of using a device, can hinder the implementation of educational interventions (Raman et al. 2015). To our knowledge, during the time of this trial, no system-level policies at any participating site impeded the use of our mobile technology.

With respect to the reminder dimension, educational interventions can be operationalized in different ways; e.g. as alerts or notifications delivered through a mobile app versus reminders arriving in the form of text messages (SMS). In retrospect, it is not possible to examine our findings by separately scrutinizing the influence of the technology dimension from that of the reminder dimension.

While nowhere near as powerful as an intrinsic motivation for learning that drives exam preparation, alerts delivered through a mobile app could act as extrinsic stimuli to nudge residents to improve study behaviour. Alerts like this are a type of messaging commonly used to remind people about tasks such as an appointment. In health services research, text messaging reminders modestly increase attendance at healthcare appointments compared to no reminders, or postal reminders (Gurol‐Urganci et al. 2013). This finding is based on moderate quality evidence from seven studies involving 5841 participants (risk ratio 1.14, 95% confidence interval 1.03 to 1.26). In addition, alerts are a relatively low-cost intervention. Again, in the context of healthcare, the NHS implemented reminders for cervical screening in London in the form of text messages (SMS), based in part on the results of a pragmatic randomized controlled trial (Huf et al. 2020). The latter trial also found that message content significantly influenced the uptake of screening for cervical cancer.

In the current trial, we found no effect of alerts on clinical case completion, consistent with the outcome of no effect on clinical knowledge. A conclusion from this finding is as follows. While repeated exposure to didactic material is important for knowledge retention, we could not show that adding alerts to engage with clinical cases is better than self-paced review alone. This is a contribution to knowledge in the following sense. Those who plan to conduct research of this type in the future will benefit by considering our findings, and the recommendations we offer below to overcome challenges to trials of educational interventions in the Family Medicine residency.

### Limitations

In the context of Family Medicine residency training, our alerts may not have had the desired effect, for several reasons. First, participants were asked to allow notifications on the app, but we did not track message delivery. It is possible some participants did not receive the alerts as intrinsic motivation was needed to modify settings to enable alerts through the app. Intrinsic motivation was also required to overcome a variety of socio-technical issues. For example, one participant who did not install the app eventually reported her mobile device lacked space to install it. Socio-technical issues such as this were idiosyncratic to participants and their specific mobile device. Second, we could not customize the timing of alert delivery or case content in relation to a participants’ work schedule/clinical rotation. Thus, some alerts were likely perceived as irrelevant or contributing to information overload and ignored. Finally, our intervention faced competition from other educational activities. For example, at one intervention site a separate study guide was promoted to residents during the time period of our trial (Rudkin and Massoud 2017).

### Strengths

Our work has several strengths. First, under real-world conditions we demonstrated the capacity to mobilize the participation of most residents from 12 university training programs nationwide. Second, we were able to collect and analyze data on clinical case completion, a mechanism to explain any improvement in the primary outcome (exam score). The absence of an effect of our intervention on clinical case completion is consistent with the observation of no difference in the primary outcome. Even as our results indicate a practically irrelevant effect of the intervention, our trial was adequately powered to detect a difference of four percentage points in certification examination score, if such a difference existed. Power was adequate as we observed just one-half the expected standard deviation in the examination scores of participants in 2019, adequately compensating for slightly lower cluster sizes than anticipated.

Integrating alerts in the residency to encourage spaced education through a mobile app would seem to be a promising avenue for future research. As such, we have attempted to clearly describe our intervention for scholars seeking to improve educational practice. Further trials should be required before deploying spaced educational interventions in the Family Medicine residency. A requirement for such research in graduate medical education should be to co-design a version of any online learning tool (such as the Family Medicine Study Guide) that remains locked until a participant signs in to enable tracking of their app use. In addition to this very important technical requirement, crafting the content of alert messages to optimally motivate learners in a graduate medical education context will likely benefit from a co-design approach.

We took a ‘hands off’ approach in this trial by not specifically asking clinician educators to promote the use of the app. Future research would benefit from organizational support to optimize the participation of this group of stakeholders.
